# Mitochondria Targeting as an Effective Strategy for Cancer Therapy

**DOI:** 10.3390/ijms21093363

**Published:** 2020-05-09

**Authors:** Poorva Ghosh, Chantal Vidal, Sanchareeka Dey, Li Zhang

**Affiliations:** Department of Biological Sciences, The University of Texas at Dallas, Richardson, TX 75080, USA; pgg130130@utdallas.edu (P.G.); cxv130930@utdallas.edu (C.V.); sxd100620@utdallas.edu (S.D.)

**Keywords:** mitochondria, metabolism, OXPHOS, heme

## Abstract

Mitochondria are well known for their role in ATP production and biosynthesis of macromolecules. Importantly, increasing experimental evidence points to the roles of mitochondrial bioenergetics, dynamics, and signaling in tumorigenesis. Recent studies have shown that many types of cancer cells, including metastatic tumor cells, therapy-resistant tumor cells, and cancer stem cells, are reliant on mitochondrial respiration, and upregulate oxidative phosphorylation (OXPHOS) activity to fuel tumorigenesis. Mitochondrial metabolism is crucial for tumor proliferation, tumor survival, and metastasis. Mitochondrial OXPHOS dependency of cancer has been shown to underlie the development of resistance to chemotherapy and radiotherapy. Furthermore, recent studies have demonstrated that elevated heme synthesis and uptake leads to intensified mitochondrial respiration and ATP generation, thereby promoting tumorigenic functions in non-small cell lung cancer (NSCLC) cells. Also, lowering heme uptake/synthesis inhibits mitochondrial OXPHOS and effectively reduces oxygen consumption, thereby inhibiting cancer cell proliferation, migration, and tumor growth in NSCLC. Besides metabolic changes, mitochondrial dynamics such as fission and fusion are also altered in cancer cells. These alterations render mitochondria a vulnerable target for cancer therapy. This review summarizes recent advances in the understanding of mitochondrial alterations in cancer cells that contribute to tumorigenesis and the development of drug resistance. It highlights novel approaches involving mitochondria targeting in cancer therapy.

## 1. Introduction

**Figure 1:**
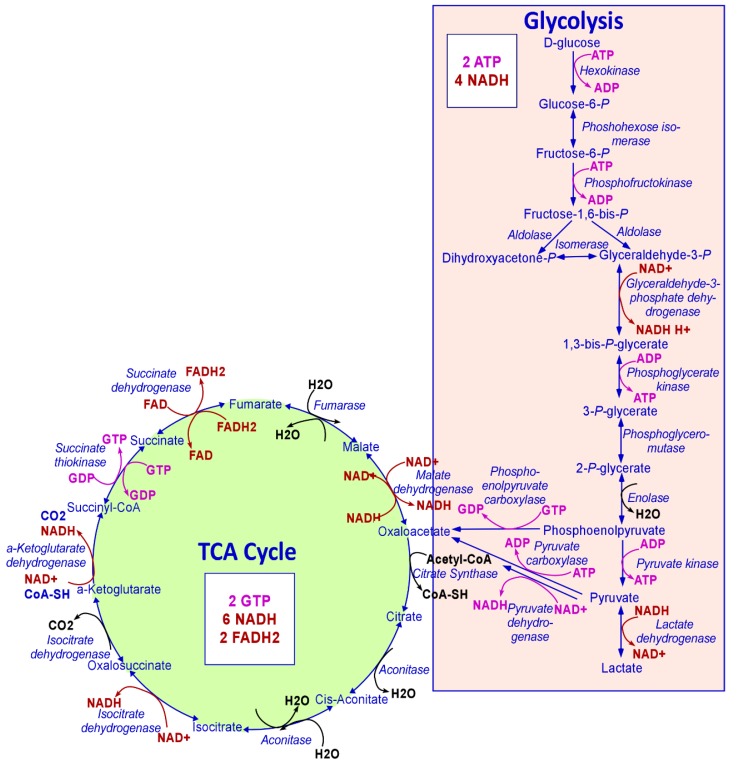
The metabolic steps of glycolysis and TCA cycle. Every step of glycolysis and the TCA cycle are shown. The NAD+/NADH and FAD/FADH2 generated or utilized are shown in red. The ATP/GTP synthesized and consumed is shown in pink. The numbers of ATP, GTP, NADH, and FADH2 generated when one molecule of glucose is consumed following glycolysis, and the TCA cycle are also shown.

Mitochondria play many essential roles in eukaryotic cells. Firstly, mitochondria are the principal location for adenosine triphosphate (ATP) production to satisfy the bioenergetic needs of the cell. Several carbon sources are utilized to produce ATP, including pyruvate generated from glycolysis, glutamine, and fatty acids. These then enter the tricarboxylic acid (TCA) cycle in the mitochondrial matrix to generate NADH and FADH2, to transfer their electrons to the electron transport chain (ETC) embedded in the inner mitochondrial membrane [1], a process known as oxidative phosphorylation (OXPHOS) (Figure 1). About 90% of cellular ATP is generated in mitochondria through this OXPHOS pathway. Secondly, mitochondria operate as a central hub of both catabolic and anabolic reactions that allow high metabolic adaptation of cancer cells. In this context, acetyl coenzyme A (acetyl-CoA) is condensed with oxaloacetate by citrate synthase (CS, the first enzyme of the TCA cycle) in the mitochondria, generating citrate and free CoA. Unlike acetyl-CoA, citrate can be exported to the cytosol through SLC25A1, followed by the regeneration of oxaloacetate and acetyl-CoA by ACLY. The export of citrate from mitochondria to the cytosol generates the need for the replenishment of the TCA cycle intermediates that regenerate oxaloacetate [2]. Moreover, intermediates in the TCA cycle are used in macromolecule synthesis to meet the biosynthetic needs of cell growth and proliferation. Mitochondria are also involved in other processes such as heme biosynthesis, which is indispensable for cellular respiration, energy metabolism, and cell survival [3]. Mitochondria alter their bioenergetic and biosynthetic functions to meet the metabolic demands of the cell and continuously communicate their fitness to the rest of the cell [1].

Emerging evidence suggests that cancer is primarily a mitochondrial metabolic disease [4,5,6,7]. Cancer cells undergo metabolic rewiring to accommodate their increased bioenergetic needs, however, this rewiring may differ within tumors. Tumors display metabolic heterogeneity within themselves. Tumor cells metabolize various fuels like glucose, lactate, pyruvate, hydroxybutyrate, acetate, glutamine, and fatty acids at much higher rates than normal cells. Differences in the localization of biochemical pathways within subcellular compartments, and the transfer of catabolites among these, add to the complexity of the metabolic profile of tumors. This metabolic heterogeneity enables tumor cells to generate ATP, maintain the redox balance, as well as to provide resources for various biosynthetic processes essential for cell survival, growth, and proliferation [4]. This metabolic flexibility is, in part, attributable to molecules such as acetyl-CoA, which is a central metabolic intermediate. Acetyl-CoA controls key cellular processes, including energy metabolism, mitosis, and autophagy. It determines the balance between cellular catabolism and anabolism by simultaneously operating as a metabolic intermediate and as a second messenger [2].

In addition to altered metabolism, cancer cells also exhibit altered mitochondrial function in general, including mitochondrial transport, dynamics, and response to oxidative stress. In this review, we focus on the most frequent aberrations in mitochondrial functions and strategies to target the aforementioned aberrations. We also highlight the importance of heme, an important player in mitochondrial homeostasis and tumor progression.

## 2. Mitochondrial Function Is Altered in Diverse Cancer

Despite being highly diverse, cancer cells display stereotypical traits, known as hallmarks. In the majority of these hallmarks, mitochondria play key roles [5]. Mitochondrial transformations, including bioenergetics, metabolism, and fission-fusion dynamics, play an important role in tumorigenesis. Altered bioenergetics help cancer cells meet their energy demand by generating ATP, via the ETC, while altered mitochondrial metabolism supports these rapidly proliferating cells by providing them with building blocks. The flexibility that mitochondria bestow tumor cells allows for their survival in the face of adverse environmental conditions such as starvation, as well as during chemotherapeutic and targeted cancer treatments [6]. Thus, understanding the mechanisms of mitochondrial function during tumorigenesis will be critical for the next generation of cancer therapeutics.

### 2.1. Upregulation of OXPHOS in Cancer Cells Drives Cellular Bioenergetics

With an increasing amount of new experimental data, the misconception that malignant cells satisfy their bioenergetic and anabolic needs mostly via aerobic glycolysis has been overturned. It is now widely accepted that tumors form and develop under the influence of mitochondrial metabolism on all steps of oncogenesis, i.e., malignant transformation, tumor progression, and response to treatment [7]. In fact, various cancer cells depend on OXPHOS to promote tumorigenic potential [8,9]. For example, a study analyzing glioma cells found that oxidative substrates such as pyruvate and lactate were able to rescue glioma cells under low glucose conditions [10]. Another study analyzing MCF-7 cells showed that 80% of ATP is generated by oxidative metabolism and that higher rates of glucose consumption are not necessarily linked to an increase in glycolytic rate [11]. Moreover, a study analyzing drug-resistant cancer cells found that these cells become reliant on mitochondrial OXPHOS for survival. Treating these cells with inhibitors of complex I of the ETC prolonged survival and tumor burden in murine xenograft models [12].

Invasive cancer cells favor increased mitochondrial respiration; utilizing PGC1α (peroxisome proliferator-activated receptor-gamma coactivator) to enhance mitochondrial biogenesis and OXPHOS. The knockdown of PGC1α also causes a significant reduction in circulating cancer cells. These results demonstrate that PGC1α-mediated mitochondrial biogenesis and mitochondrial respiration is crucial for metastatic cancer cells [13]. A study analyzing mitochondrial biogenesis and drug resistance found that patients whose tumor biopsies had a higher expression of mitochondrial biogenesis before treatment had worse overall survival [14]. Interestingly, the authors showed that melanoma cells with higher mitochondrial biogenesis before treatment with MAPK (mitogen-activated protein kinase) inhibitors became sensitive to treatment by downregulating mitochondrial biogenesis. Contrarily, melanoma cells that had lower mitochondrial biogenesis before treatment with MAPK inhibitors became resistant to treatment by upregulating mitochondrial biogenesis [14]. Metastatic colorectal cancer cells, such as SW620 cells, have been found to express higher levels of TFAM and mitochondrial DNA (mtDNA) than primary SW480 cells. These metastatic cancer cells also expressed lower levels of glycolytic enzymes and lactate production rates, but higher rates of oxygen consumption [15]. OXPHOS is also upregulated in epithelial breast cancer cells, which allows them to produce high levels of ATP [16]. Furthermore, AMPK (AMP-activated protein kinase), an energy sensor and metabolic switch, is known to be activated by LKB1 (liver kinase B1) under energy stresses. The activation of AMPK induces the expression of p21/WAF1. This increase of p21/WAF1 may be the underlying mechanism of growth arrest when cancer cells are exposed to energy stresses and ATP depletion [17].

Growing evidence also suggests that autophagy supports the metabolic plasticity of cancer cells. The degradation of proteins, organelles, and lipids can generate new metabolic substrates and maintain bioenergetic needs for cells [18]. Mitophagy, or the degradation of dysfunctional and obsolete mitochondria, is known to be involved in tumor progression. In melanoma cells, mitophagy is needed to prevent the accumulation of damaged mitochondria, which allows cells to adjust to the metabolic and nutrient stress from the microenvironment [19].

Heterogeneity of cancer cells due to the presence of functionally diverse subpopulations like cancer stem cells (CSCs) is mainly responsible for disease progression and therapy failure. OXPHOS is the main energy source in CSCs. Cancer cells are usually surrounded by noncancerous host tissue called stroma, which plays an important role in tumor growth and metastasis [20]. A variety of metabolites released by stromal cells can be used by OXPHOS-dependent CSCs to adapt to the changing conditions of the tumor microenvironment and utilize it to fuel the TCA cycle [21].

Stromal cells undergo aerobic glycolysis and produce energy-rich nutrients such as lactate. This lactate is used by cancer cells that prefer lactate as the fuel source in comparison to glucose. While utilizing lactate for respiration, these cancer cells produce reactive oxygen species (ROS), which enters the membrane of surrounding stromal cells such as fibroblasts. The resulting oxidative damage from ROS generation causes a metabolic switch from oxidative metabolism to glycolytic metabolism in these fibroblasts. These fibroblasts then release lactate and ketone bodies, which in turn, are taken up by the oxidative tumor cells as fuel. This mechanism explains how cancer cells survive during metastasis and how their growth is fueled [22,23].

Together, these studies suggest that cancer cells often shift from anaerobic respiration to oxidative phosphorylation in response to increased energy needs. This upregulation of OXPHOS is a vulnerability that can be exploited with OXPHOS inhibitors. It is important to note that for OXPHOS to be upregulated, there needs to be constant feedback between OXPHOS and the TCA cycle, the central metabolic pathway of the mitochondria.

### 2.2. Mitochondrial Metabolism Is Reprogrammed in Cancer Cells

In addition to providing the major source of energy for tumor progression, mitochondria are indispensable to cancer cells due to their ability to process metabolic intermediates from several pathways via the TCA cycle, in turn, providing building blocks for anabolism. The regulation of the TCA cycle and its constant feedback with OXPHOS is important for the maintenance of cancer cells. The TCA cycle constitutes an epicenter in cell metabolism because multiple substrates can feed into it.

Several enzymes of the TCA cycle are often mutated or deregulated in human cancers, including aconitase (also known as aconitate hydratase, AH), isocitrate dehydrogenase (IDH), fumarate hydratase (FH), succinate dehydrogenase (SDH), and KGDHC [24,25]. Moreover, metabolites in the TCA cycle are also involved in controlling chromatin modifications, DNA methylation, and post-translational modifications of proteins to alter their function.

Todisco et al. summarized the role of the TCA cycle rewiring in hepatocellular carcinoma. They reported gene expression and activity dysregulation of enzymes relating to the TCA cycle, glutamine metabolism, malate/aspartate, and citrate/pyruvate shuttles [26]. They also focused on the link between NF-κB-HIF1 transcriptional factors and TCA cycle reprogramming [26]. Dekker et al. found significant changes in the abundance of enzymes associated with aerobic glycolysis, glutamate metabolism, and the TCA cycle in IDH1 mutant gliomas [27]. Specifically, the enzymes involved in the metabolism of glutamate, lactate, and the conversion of α-ketoglutarate were significantly increased. There was also increased expression of the bicarbonate transporter (SLC4A4), suggesting that a mechanism to prevent intracellular acidification (due to increased glycolysis) is active. This kind of vast metabolic rewiring occurs to preserve TCA-cycle activity in IDH1-mutant gliomas [27].

The excessive conversion of glucose to lactate, as well as the export of citrate from mitochondria to the cytosol by the dicarboxylate antiporter solute carrier family 25 (mitochondrial carrier; citrate transporter) member 1 (SLC25A1), drives tumor cells to use anaplerotic reactions to replenish TCA cycle intermediates; this is largely achieved through increased glutaminolysis. Mitochondrial glutamate is converted into α-ketoglutarate catalyzed by glutamate dehydrogenase 2 (GLUD2), or glutamic-oxaloacetic transaminase 2 (GOT2). This α-ketoglutarate undergoes reductive carboxylation within the TCA cycle to generate citrate. Citrate is then exported to the cytosol via SLC25A1 and converted into oxaloacetate and acetyl-CoA by ATP citrate lyase (ACLY), which is eventually utilized in the synthesis of fatty acids and steroids. Therefore, progressing tumors display the ability to process glutamine oxidatively for energy production via the Krebs cycle and the ETC [2,7,28,29].

Elevated glutamine transport and glutaminolysis enable numerous cancer types, including myeloma and glioma tumor cells, to derive large portions of their energy and macromolecules [30,31]. Enzymes like ACLY and ACC, that regulate lipid synthesis, are often overexpressed in multiple types of cancers. ACLY is upregulated in NSCLC, breast cancer, and cervical cancer, etc. [32,33,34], while ACC is upregulated in NSCLC and hepatocellular carcinoma [35,36].

### 2.3. Altered Mitochondrial Metabolism Is Accompanied by Oxidative Stress in Cancer Cells

Metabolic aberrations in cancer cells lead to the accumulation of ROS, which in turn causes biomolecular damage and cell death when present at high levels [37,38]. During metabolic processes, electrons released from the mitochondrial ETC are captured by molecular oxygen to generate superoxide anions, which leads to the production of ROS [37,39,40]. These ROS, in turn, can cause DNA damage, interact with surface and intracellular receptors, as well as signaling pathways. They can also disrupt processes related to proliferation, apoptosis, and angiogenesis, which are important aspects of cancer progression [41,42]. In many cancers, ROS-mediated DNA damage promotes the initiation of carcinogenesis and the malignant transformation of cells. Also, ROS can facilitate cell survival and cancer progression. However, excessive ROS causes cytochrome.c release into the cytoplasm and triggers programmed cell death [43]. High levels of ROS pose a severe risk to mitochondrial integrity and, therefore, cell viability. Therefore, cancer cells need to strike a balance between various mechanisms, such as mitochondrial biogenesis and mitophagy, to maintain mitochondrial fitness [44].

There are many players involved in the oxidative stress adaptation of cancer cells. For example, in breast cancer cells, alterations in the genome are produced due to ROS generated by estrogen-induced oxidative stress. Also, oxidative stress adaptation resulting from the downregulation of estrogen receptors leads to aggressive growth potential and resistance to endocrine therapy [45,46]. In normal cells, ROS levels are tightly controlled by an inducible antioxidant program that responds to cellular stresses, which are regulated by the transcription factor NRF2 (also known as Nfe2l2) and its repressor protein KEAP1. Identification of somatic mutations that disrupt the Nrf2–Keap1 interaction to stabilize NRF2 point to enhanced ROS detoxification and protumorigenic functions of NRF2. Oncogenes such as Kras, Braf, and Myc are known to actively suppress ROS. Also, genetic targeting of the NRF2 pathway impairs K-Ras G12D-induced proliferation and tumorigenesis in vivo [47]. Studies have shown that disrupting antioxidant mechanisms triggers ROS-mediated cell death in a variety of cancer cell types [48]. The NRF2 system, including upregulation of the heme degradation enzyme HO-1, plays a pivotal role in cellular adaptation to oxidative stress. Activation of this system in normal cells is essential in the prevention of carcinogenesis [49]. However, in cancer cells, HO-1 is constantly activated to favor tumor growth, metastasis and invasion, and resistance to therapy, and therefore, leads to a poor therapeutic outcome [50]. There are other possible mechanisms by which increased ROS leads to increased tumor growth and metastasis. Subpopulations of cancer cells are now known to upregulate protective pathways, such as the mitochondrial unfolded protein response (UPRmt), which maintain ROS and improve mitochondrial fitness. This is due to the persistent activation of cytoprotective mechanisms resulting from constant mitochondrial stress.

Consequently, these cells are resistant to oxidative stress and have increased metastatic capacity [44]. Dysregulated metabolism and oxidative stress are common features of cancer, which can, in turn, regulate mitochondrial dynamics. Cancer patients have tumors of different origins and stages. It is important to understand that different metabolic states under these pathology conditions lead to altered mitochondrial dynamics. Therefore, mitochondrial metabolism and mitochondrial dynamics should be studied together in a context-dependent manner.

### 2.4. Cancer Cells Exhibit Altered Mitochondrial Dynamics

Normal mitochondrial function is maintained by continuous fusion and fission. As expected, recent studies have shown that proteins involved in the fusion-fission machinery regulate the intrinsic apoptotic pathway [51]. For example, genes involved in the regulation of mitochondrial dynamics are amplified in human cancer cells and inhibiting the fission mediating GTPase DRP1 (dynamin-related protein 1), is shown to induce apoptosis [52]. The knockout of DRP1 in pancreatic cancer cells has been shown to decrease oxygen consumption rates and ATP production levels, leading to reduced growth in vivo [53]. Lung adenocarcinoma tissues also express high levels of DRP1 and lower levels of MFN-2 (mitofusin 2) when compared to adjacent normal lung.

Additionally, overexpression of MFN-2 in the NSCLC A549 cell line leads to a dose-dependent decrease in proliferation [54].

DRP1 dysregulation can act on different phases of the cell cycle and helps in cell proliferation. For example, DRP1 can inhibit p53 and promote progression into the cell cycle [55,56]. Epithelial ovarian cancer cells co-express DRP1 with cell cycle genes, and 55% of these co-expressed genes are involved in mitotic transition [57]. Inhibiting DRP1 causes replication stress and a delay in G2 to M transition in the cell cycle. This replication stress is caused by a hyperfused mitochondrial structure and an unscheduled cyclin E expression in the G2 phase. This induces an ATM-dependent G2-M transition cell cycle checkpoint. [58]. Furthermore, a study on invasive breast cancer cells found that silencing DRP1 suppressed metastatic abilities by inhibiting lamellipodia formation [59]. Similarly, DRP1 expression was upregulated in hypoxic glioblastoma U251 cells and inhibition of DRP1 attenuated hypoxia-induced mitochondrial fission and migration. These studies suggest that DRP1 might be a potential therapeutic target [60]. However, a study analyzing the U20S cell line found that chemotherapy-induced apoptosis can lead to proteasomal degradation of MFN-2, causing mitochondrial fragmentation and apoptotic cell death [61,62].

The subcellular distribution of mitochondria has been implicated in cancer cell proliferation, metastasis, and response to therapy. Similarly, metastatic traits of cancer cells have been associated with enhanced mitochondrial trafficking. [63]. Mitochondrial trafficking in cancer involves many proteins like Rho GTPases MIRO1/2, trafficking adapter proteins TRAK1/2 that bind to kinesin1/3, etc. TRAK1 is upregulated in gastric and colorectal cancers and can serve as a promising diagnostic marker [64]. MIRO1 is known to play an important role in the mitochondrial distribution along with microtubule networks. Particularly, in cancer cells, interfering with MIRO1 can reduce migratory abilities [65]. Also, myosins (MYOs) or actin-based motors play an important role in cellular motility and are largely expressed in cancer [66,67]. Targeting altered mitochondrial dynamics and trafficking holds immense potential as a therapeutic strategy for cancer, given the reliance of cancer cells on these for their proliferation and metastasis.

### 2.5. Elevated Heme Levels Promote Proliferation and Tumorigenic Potential of Cancer Cells

Heme, also known as iron protoporphyrin IX, is an essential iron-containing molecule that can be synthesized at a basal level by human cells [68]. This biosynthetic pathway requires eight enzymes that convert glycine and succinyl-CoA to heme. Additionally, dietary heme can be acquired through red meat intake. Diseases such as anemia, porphyrias, and Alzheimer’s disease have been linked to a deficiency of heme [69]. Furthermore, epidemiological studies have suggested that high heme intake is associated with an increased risk of several types of cancers, including colorectal, lung, pancreatic, breast, and esophageal cancer. In a study of approximately 500,000 individuals, the highest quantile of processed meat intake had a 16% higher risk of lung cancer [69,70,71,72]. To determine whether dietary heme intake from red meat caused this increased risk of colon cancer, another study analyzed the effect of heme on the proliferation of colon cancer cells. They determined that colonic epithelial proliferation in heme-fed rats was significantly increased compared to control rats [73]. Dietary heme in meat has also been found to promote pre-neoplastic lesions in the colon [74].

Heme regulates various processes, including those relating to oxygen utilization and metabolism [75]. It serves as a prosthetic group or cofactor in enzymes that transport and use oxygen, and therefore, it is a central molecule in mitochondrial OXPHOS [76]. Complex II, III, and IV of the ETC require heme to function [22]. In addition to the role of heme in the ETC, the trafficking of ADP and ATP between mitochondria and cytosol can be controlled by heme. The translocase involved in this trafficking is the adenine nucleotide transporter (ANT). ADP uptake can be competitively inhibited by heme and its precursors, suggesting that ANTs contribute to heme biosynthesis by transporting heme precursors into the mitochondria. Particularly, ANT2 isoform, which has been postulated to contribute to the metabolic adaptations of tumor cells, is assumed to be regulated by heme [77,78]. The critical role of heme in mitochondrial respiration and ADP/ATP exchange presumably explains how heme plays a pivotal role in fueling the proliferation of tumor cells.

**Figure 2:**
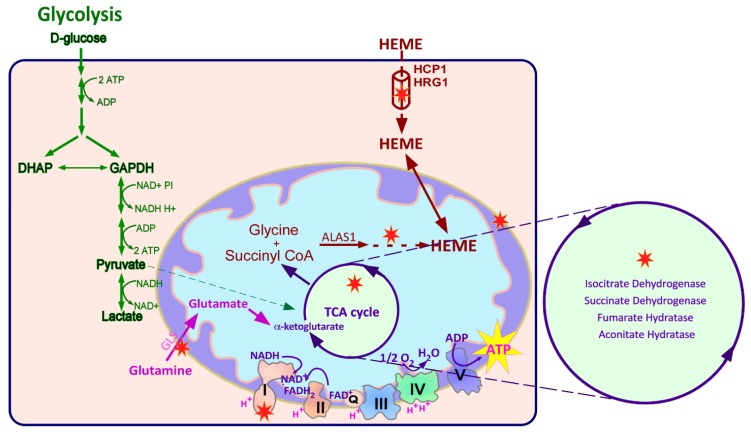
Cancer cells exhibit altered metabolism that provides many potential targets for cancer therapy. Shown here are some altered metabolic processes that cancer cells rely on to satisfy their increased bioenergetic demands, including elevated glycolysis (reactions represented by green arrows), oxidative phosphorylation (reactions in TCA cycle and the electron transport chain represented with purple arrows), glutaminolysis (reaction represented by pink arrows), and elevated heme levels. These metabolic aberrations provide numerous targets for cancer therapy, which are represented using red five-pointed stars. In cancer cells, enzymes of the TCA cycle, such as aconitase (aconitate hydratase, AH), fumarate hydratase (FH), isocitrate dehydrogenase (IDH), and succinate dehydrogenase (SDH) are often mutated or deregulated. These mutated enzymes can be targeted for cancer therapy. Also shown is elevated glutamine transport and glutaminolysis (conversion of glutamine to glutamate by glutaminase or GLS) to support the excessive conversion of glucose to lactate that drives tumor cells to use anaplerotic reactions to replenish TCA cycle intermediates. The inhibition of glutaminolysis may prove to be an effective therapeutic strategy for the treatment of cancer. Heme synthesis involving the enzyme ALAS1 (aminolevulinate synthase), the rate-limiting step of heme biosynthesis, is elevated in NSCLC cells (reactions represented with brown dotted arrows). Also elevated is the process of heme uptake by heme transport proteins, HCP1 (Heme carrier protein 1) and HRG1 (Heme responsive gene 1), represented here as the brown cylinder. Both processes contribute to elevated levels of heme in NSCLC cells. Elevated heme via increased heme synthesis and/or uptake is exhibited by cancer cells to fuel elevated OXPHOS in NSCLC. Limiting heme availability by inhibiting heme synthesis or uptake can also be a novel and effective therapeutic strategy that targets OXPHOS in NSCLC. Also shown is the electron transport chain, including complexes I, II, III, IV, V, and coenzyme Q or ubiquinone (shuttles electrons between complexes I or II, and complex III). Proper functioning of the ETC is required for energy generation via OXPHOS. These ETC complex proteins can also be targeted for cancer therapy.

Recent studies in the authors’ laboratory have demonstrated that the levels of heme flux are significantly elevated in lung tumors [79]. Using various representative NSCLC cell lines, it was demonstrated that heme biosynthesis and uptake were significantly increased in the NSCLC cell lines compared to nontumorigenic cells. This increase in heme biosynthesis and uptake correlated with an elevation of the heme biosynthesis enzyme, ALAS1, and the heme uptake protein SLC48A1, as summarized in Figure 2 [79]. Overexpressing ALAS1 or SLC48A1 also promoted intensified levels of oxygen consumption and ATP generation, which caused an increase in the tumorigenic potential of NSCLC cells. Overall the data revealed that heme plays a critical role in the progression of lung cancer.

## 3. Targeting OXPHOS and Heme Inhibits Growth and Progression in Multiple Cancers

There are a plethora of drugs that target mitochondria, and it is becoming increasingly important to elucidate the mechanisms involved in these effects. Proper functioning of the ETC is required to support OXPHOS activity and ATP synthesis, which is essential for tumorigenesis. ETC inhibitors such as metformin, tamoxifen, α-tocopheryl succinate (α-TOS), and 3-bromopyruvate (3BP) act via disrupting the function of respiratory complexes of the ETC and inducing high levels of ROS to kill cancer cells [80]. Targeting these drugs directly to the mitochondria of cancer cells is a novel approach that is being investigated for cancer therapy.

Many therapeutic agents such as MitoTam, a novel mitochondrial-targeted derivative of tamoxifen, inhibits complex I of the ETC, increases ROS production, thereby causing cell death in breast cancer cells [81]. Also, MitoVES, a mitochondrially targeted analog of vitamin E succinate, inhibits complex II of ETC, thereby reducing tumor growth by causing apoptotic cell death in breast, colorectal, and lung cancer [82]. Other therapeutic strategies that directly target respiratory complex I, the first and rate-limiting enzyme of OXPHOS, are also gaining momentum as a potential therapeutic approach. In an interesting study by Qi Huang et al., a small molecule inhibitor ME-344 was able to target not just complex I, but several other signaling pathways that lead to cell death induction associated with mitochondrial permeability transition in colorectal cancer [83]. Two molecules ME-143 and ME-344 significantly inhibited the ability of complex I to oxidize NADH, preventing electron flux through the OXPHOS complexes. ME-344 inhibition of complex I also caused the generation of ROS, leading to the translocation of Bax to the mitochondrial outer membrane, which induced mitochondrial permeability transition (MPT), resulting in the release of pro-apoptotic molecules [84]. A major metastasis promoting protein, S100 calcium-binding protein A4 (S100A4), was shown to confer metabolic plasticity that drives invasion and metastasis in NSCLC [85]. Reduction in S100A4 levels was shown to reduce oxygen consumption rates, mitochondrial function, and ATP synthesis, thereby shifting cell metabolism to glycolysis. S100A4 silencing inhibited mitochondrial complex I activity, reduced cellular ATP levels, and decreased invasive and metastasic properties as well as tumor growth in vivo, suggesting a link between mitochondrial function and S100A4. This idea is supported by the fact that niclosamide, which is known to inhibit mitochondrial oxidative phosphorylation, also targets S100A4 in lung cancer and colon cancer [85].

Tocotrienols (γ-T3), a subgroup of Vitamin E interacts with mitochondrial electron transfer chain NDUFB8 (a subunit of complex I) and SDHB (a subunit of complex II) to inhibit OXPHOS and leads to ROS production [86]. The biguanide metformin, a commonly used drug to treat type 2 diabetes, is now being repurposed for the treatment of cancers. Metformin is known to directly act on mitochondria to limit TCA cycle activity and OXPHOS, as demonstrated in isolated mitochondria as well as in intact cells [87]. It directly inhibits mitochondrial complex I (NADH dehydrogenase), resulting in decreased mitochondrial respiration [88,89]. It not only reduces mitochondrial respiration but is also known to inhibit lipid metabolism, glucose metabolism, methionine cycle, folate cycle, nucleotide synthesis, and mTOR signaling [90,91,92,93]. Therefore, metformin has immense potential as an anti-cancer therapy.

Given the role of elevated heme metabolism for the tumorigenic functions of NSCLC cells, limiting heme availability may be another effective strategy to inhibit lung tumor growth and progression. Mammalian cells access heme via two means; they synthesize heme de novo and uptake extracellular heme using the heme uptake/transport machinery. Inhibiting heme synthesis and/or inhibiting heme uptake can be used to limit heme availability to NSCLC cells (Figure 2). Various agents, such as succinyl acetone (SA), an inhibitor of heme biosynthesis, have been tested for their anti-cancer efficacy [79].

One of the other agents that inhibits heme synthesis is cyclopamine tartrate (CycT). Cyclopamine was initially identified as an inhibitor of smoothened (SMO), a G protein-coupled receptor that positively regulates hedgehog (Hh) signaling. Since then, an array of SMO antagonists has been developed and tested for cancer treatment. CycT is a more potent, water-soluble form of cyclopamine. CycT inhibits mitochondrial respiration in an array of NSCLC cell lines [94]. CycT inhibits proliferation, colony formation, invasion, and migration of NSCLC cells. It also delays the growth and progression of NSCLC in subcutaneous as well as orthotopic lung tumor xenografts. The anti-cancer activity of CycT is not solely attributable to it being a Hh signaling inhibitor. Rather, CycT diminishes the levels of proteins involved in heme biosynthesis, uptake, transport, and selectively inhibits heme synthesis. CycT inhibits OXPHOS in NSCLC cells and diminishes the levels of heme- and non-heme-containing subunits of OXPHOS complexes in lung xenograft tumors [95]. Therefore, CycT can be used effectively to limit heme synthesis and OXPHOS, thereby inhibiting NSCLC growth and progression.

Many Gram-negative bacteria secrete small hemophores to bind and acquire heme from solutions or heme-containing proteins [96]. Amongst these is the well-characterized bacterial hemophore HasA, secreted by Yersinia pestis [97]. Various heme sequestering peptides (HSPs) were engineered in the authors’ lab by mutating HasA. HSP2 is one of these peptides with Q32H and Y75M mutations. HSP2 is very effective at inhibiting heme uptake in NSCLC cells. It inhibits oxygen consumption and tumorigenic functions in NSCLC cells. HSP2 inhibits the growth and progression of NSCLC subcutaneous and orthotopic lung tumor xenografts. It also reduces the levels of various OXPHOS related proteins in tumor tissues [79].

## 4. Targeting the TCA Cycle and Glutamine Metabolism for Cancer Therapy

The TCA cycle is a bioenergetic hub for metabolism, biosynthesis, and redox state balance. Therefore, inhibiting various aspects of altered bioenergetic metabolism are being explored as therapeutic strategies for the treatment of cancer. Mutations in IDH1 and IDH2 (Isocitrate dehydrogenase) genes are found in different cancers, including glioblastoma and acute myeloid leukemia (AML) [25,98]. Therapeutic agents inhibiting mutant IDH1 and IDH2 enzyme activity are now being developed for the treatment of AML and solid tumors [98,99,100,101]. Mutations in SDH and FH have also been associated with cancer and, loss of either of these enzymes leads to vulnerabilities that may be targeted effectively [102] (Figure 2). Along with TCA cycle enzymes, oncogenes such as MYC, HIF, P53, and RAS, regulate the metabolic phenotype of tumors and play an important role in regulating the TCA cycle in cancer cells [103]. These pathways are also being explored as therapeutic targets for cancer therapy [104,105,106,107].

Glutamine is used as a fuel in many different types of cancer to supply nutrients and biosynthesis precursors for their growth. Therefore, inhibition of glutaminolysis can also prove to be an effective therapeutic strategy for the treatment of cancer. Initially, glutamine analogs were being considered as potential therapeutic agents to treat cancers, however, these trials were discontinued due to severe toxicity issues [108,109]. Many efforts are now focused on inhibiting glutaminase (GLS). One of these inhibitors is CB-839, which is in phase I/II clinical trials and is known to display potent anti-tumor effects [110,111,112,113]. In various cancers, including chemotherapy-resistant brain, pancreatic, and breast cancer, 968, an inhibitor of GAC, a shorter isoform of glutaminase, also has potent antitumor effects. [114,115]. Aminooxyacetate (AOA), an inhibitor of transaminases, was shown to cause cell growth inhibition and apoptosis in c-Myc-dependent triple-negative breast cancer [116,117]. Targeting TCA, other accessory pathways, and glutamine may prove to be an effective approach for developing cancer therapy. However, cancer cells tend to adapt and develop resistance via compensatory pathways. Therefore, a thorough understanding of the alterations in the TCA cycle in cancers would be beneficial for developing new and effective therapeutic approaches.

## 5. Targeting Aberrations in Mitochondrial Dynamics and Trafficking

Mitochondrial metabolism and oxidative stress usually regulate mitochondrial dynamics. Mitochondrial dynamics including the ratio of mitochondrial fission and fusion, as well as their morphology and distribution, in turn, regulate a variety of biological processes, including energy production. Increased fission and reduced fusion of mitochondria are usually associated with cancer [118]. Several studies have, therefore, revealed aspects of mitochondrial fission that can be targeted for cancer therapy. For example, mDIVI1, an inhibitor of mitochondrial fission protein DRP1, inhibited tumorigenic capabilities of cancer stem cells in MCF7 breast cancer cells [119]. Regulating the levels of miR-125a, an inhibitor of MFN-2, which enhances mitochondrial fission in pancreatic cancer cells, can be an effective therapeutic strategy for cancer [120]. Large tumor suppressor gene 2 (LATS2) is a regulator of cell cycle and is involved in the DNA damage response. Its overexpression was shown to activate mitochondrial fission, which promotes mitochondrial stress and causes the induction of apoptosis in lung cancer cells. Thus, targeting LATS2-associated signaling and mitochondrial fission could be an effective therapeutic strategy [121]. MARCH5, an E3 ubiquitin-protein ligase involved in the regulation of mitochondrial morphology through dynamin-related protein (DMN1L) and mitofusins, was implicated in breast cancer growth and metastasis and can be a potential target for cancer therapy [122].

Mitochondrial membrane proteins and transport can also be targeted for cancer therapy. For example, TIMM50 (translocase of the inner mitochondrial membrane 50) is involved in the regulation of the ERK-P90RSK signaling pathway. It inhibits the expression of E-cadherin, which facilitates tumor proliferation and invasion in NSCLC cells, is associated with poor prognosis, and is a potential therapeutic target for NSCLC patients [123]. Also, voltage-dependent anion channel 1 (VDAC1), located at the outer mitochondrial membrane (OMM), plays an important role in mitochondria-mediated apoptosis. The association of proteins VDAC1 and HK-II (Hexokinase), an enzyme in the glycolytic pathway, promotes the inhibition of apoptosis. Therefore, targeting this complex is another emerging approach to anti-cancer treatments [124]. Cancer cells rely on aberrations in mitochondrial fission and fusion, as well as mitochondrial transport. Targeting these aberrations could be an effective therapeutic strategy for the treatment of cancer.

## 6. Mitochondrial OXPHOS Dependence Causes Therapy Resistance in Cancer Cells

Metabolic reprogramming is a major hallmark of cancer [125]. Cancer cells exhibit elevated levels of glycolysis, and therefore, OXPHOS was believed to be downregulated in all cancers. However, there is increasing evidence of upregulated OXPHOS in many types of cancer [1,126]. Moreover, many cancers, such as NSCLC, exhibit metabolic heterogeneity within tumors [127,128]. Studies on pancreatic cancer suggest that tumors not only display metabolic heterogeneity but also have sub-populations of cancer stem cells with high metastatic and tumorigenic potential, which are OXPHOS-dependent [129]. Along with metabolic heterogeneity and poor accessibility of the drug to the tumor microenvironment, these OXPHOS dependent cancer stem cell populations are known to cause therapy resistance in cancers. Many molecularly-targeted therapies cause cancer cells to depend on OXPHOS. For example, inhibition of the protein kinase BRAF in melanomas with an activating mutation in the BRAF gene causes OXPHOS dependence via induction of PGC1α, a mitochondrial biogenesis regulator [9,130]. In a study of prostate cancer cells, a metabolic shift from aerobic glycolysis to OXPHOS dictates epithelial-mesenchymal transition (EMT), increased invasion, and development of stem-like features in docetaxel-resistant cells [131]. Cytarabine-resistant AML cells exhibit elevated OXPHOS levels, and targeting mitochondrial metabolism enhances the anti-leukemic effects of cytarabine [132]. Triple-negative breast cancer cells overexpress MYC and MCL1, which together maintain chemotherapy-resistant cancer stem cells by enhancing mitochondrial OXPHOS, increasing ROS production, and inducing HIF1α expression [133]. Despite treatment with tyrosine kinase inhibitors such as Imatinib, chronic myeloid leukemia (CML) have subpopulations of leukemic stem cells (LSCs), which cause resistance to therapy. CML stem cells require OXPHOS for their survival. Therefore, targeting these with a combination treatment of imatinib and tigecycline (an antibiotic that inhibits mitochondrial protein translation) eradicates CML LSCs [134]. In such therapy-resistant cancers, targeting OXPHOS may prove to be an effective therapeutic strategy and subsequently improve the outcome for patients.

## 7. Conclusions

Undoubtedly, there is overwhelming evidence pointing to the reliance of cancer cells on altered mitochondrial function and oxidative metabolism for their proliferation and tumorigenic functions. Cancer cells exhibit not only elevated levels of OXPHOS, but also alter mitochondrial dynamics. They also exhibit elevated levels of metabolites, such as heme, which are used to fuel OXPHOS and tumorigenesis in NSCLC, as demonstrated by recent studies from the authors’ laboratory [79]. This opens up numerous therapeutic avenues and provides a plethora of potential targets for cancer therapy. Many of these targets associated with OXPHOS, the TCA cycle, or mitochondrial fission/fusion proteins are already being investigated. Aggressive cancers become resistant to most chemotherapy drugs due to the presence of clusters of drug-resistant cancer stem cells that exhibit an altered metabolic profile. Despite the availability of many molecular targeted therapies, the therapeutic outcome for cancers has only slightly improved due to the eventual development of drug resistance among these molecularly-targeted cancers. Therefore, to treat genetically and metabolically diverse cancers, it is essential to investigate novel therapeutic avenues that are effective against a wide variety of cancers. Targeting OXPHOS or bioenergetics of cancer cells via the limitation of key factors like heme may prove to be one such promising approach [79,95]. Nevertheless, a more thorough understanding of the impacts of limiting heme on cancer is required not only to design more effective therapeutic strategies but also to aid currently available therapies for the treatment of therapy-resistant cancer.

## Abbreviations

ACC	acetyl coenzyme A carboxylase
ACLY	ATP citrate lyase
AOA	aminooxyacetate
AML	acute myeloid leukemia
AMPK	AMP-activated protein kinase
ANT	adenine nucleotide transporter
ATP	adenosine Triphosphate
AH	aconitate hydratase
CML	chronic myeloid leukemia
CycT	cyclopamine tartrate
DMN1L	dynamin 1-like related protein
DRP1	dynamin-related protein
EMT	epithelial-mesenchymal transition
ETC	electron transport chain
FH	fumarate hydratase
HIF1	hypoxia inducing factor -1
HK-II	hexokinase-II
HSPs	heme sequestering peptides
IDH	isocitrate dehydrogenase
KEAP1	kelch Like ECH Associated Protein 1
KGDHC	alpha-ketoglutarate dehydrogenase
LATS2	large tumor suppressor gene 2
LKB1	liver Kinase B1
MAPK	mitogen-activated protein kinase
MCL1	myeloid Leukemia Cell Differentiation Protein
MFN-2	mitofusin 2
MYO	myosin
NF-κB	nuclear factor kappa-light-chain-enhancer of activated B cells
NRF2	nuclear factor erythroid 2–related factor 2
NSCLC	non-small cell lung cancer
OXPHOS	oxidative phosphorylation
OMM	outer mitochondrial membrane
p21/WAF1	cyclin-dependent kinase inhibitor 1
PGC1α	peroxisome proliferator-activated receptor gamma coactivator 1 alpha
ROS	reactive oxygen species
SA	succinyl acetone
SDH	succinate dehydrogenase
TCA	tricarboxylic acid cycle
TFAM	transcription factor A, mitochondria
TIMM50	translocase of the inner mitochondrial membrane 50
TRAK	trafficking Kinesin Protein
VDAC	voltage-dependent anion channel 1
